# Can Oral Fluids Replace Nasal Swabs in Swine Influenza A Virus (swIAV) PCR Diagnostics?

**DOI:** 10.3390/pathogens14080808

**Published:** 2025-08-14

**Authors:** Aleksandra Woźniak, Piotr Cybulski, Pia Ryt-Hansen, Lars Erik Larsen, Kinga Biernacka, Dagmara Miłek, Tomasz Stadejek

**Affiliations:** 1 Department of Pathology and Veterinary Diagnostic, Institute of Veterinary Medicine, Warsaw University of Life Sciences-SGGW, 02-776 Warsaw, Poland; dagmara_milek@sggw.edu.pl; 2 Goodvalley Agro S.A., Dworcowa 25, 77-320 Przechlewo, Poland; piotr.cybulski@goodvalley.com; 3 Department of Veterinary and Animal Sciences, University of Copenhagen, 1870 Frederiksberg, Denmark; piarh@sund.ku.dk (P.R.-H.); lael@sund.ku.dk (L.E.L.); 4 Division of Veterinary Epidemiology and Economics, Institute of Veterinary Medicine, Warsaw University of Life Sciences-SGGW, 02-776 Warsaw, Poland; kinga_biernacka@sggw.edu.pl

**Keywords:** oral fluid, IAV, nasal swabs, subtyping, influenza, pig

## Abstract

The diagnosis of swine influenza A virus (swIAV) has to involve laboratory detection, as the clinical signs are not pathognomonic. Nasal swabs (NSs) have been the preferred sample material for swIAV PCR diagnostics, but oral fluid (OF) is a convenient alternative material. In this study, NSs and OFs from 35 Polish swine herds were collected and tested with real-time RT-PCR in order to assess swIAV circulation patterns in Poland and improve protocols for efficient, non-invasive and cost-effective swIAV surveillance in pig farms. The study showed that the swIAV RNA was detected in 65.7% of the tested farms. In total, 21.2% of NS pools and 48.6% of OF samples were positive for swIAV. The Ct values in NS pools and OFs were similar (*p* > 0.05), but a significant reduction (*p* < 0.05) in swIAV prevalence in NSs was observed in nursery pigs from farms applying swIAV vaccinations. Successful subtyping was achieved more effectively with OFs compared to NSs, and the H1avN2 was most prevalent subtype detected. The results emphasized that OF can be useful for monitoring swIAV and subtyping. However, OFs cannot replace NSs, which were more useful in the assessment of the effect of swIAV vaccinations in nursery pigs.

## 1. Introduction

Influenza A virus (IAV) is a virus with a segmented, single-stranded RNA genome, which belongs to the family *Orthomyxoviridae* (genus *Alphainfluenzavirus*) [1]. Swine influenza A virus (swIAV) is divided into subtypes based on the antigenicity of its hemagglutinin (HA) and neuraminidase (NA) proteins [1]. Pigs play a significant role in IAV evolution and influence viral distribution on a global scale [2]. SwIAV replicates in the respiratory tract; thus, virus transmission occurs mainly via the respiratory route [3]. In pigs, clinical signs, such as fever, lethargy and respiratory distress, including nasal discharge, coughing and sneezing, last for a few days [4]. Influenza causes significant economic losses because of fever and weight loss in growing pigs or reproductive failure in breeding sows [1]. Moreover, swIAV is considered one of the major pathogens involved in porcine respiratory disease complex (PRDC) [1].

The immune response to swIAV infection is rapid and highly efficient, and the virus is usually eliminated within one week of infection [5]. However, due to the concurrent circulation of antigenically unrelated strains in a herd and maternally derived antibodies (MDAs), pigs can be prolonged shedders, re-exposed, re-infected, and eventually exhibit clinical signs of infection with a diverse swIAV strain. Along with management changes to limit viral spread, sow vaccination is the main measure to control influenza, which protects them and their progeny from severe clinical disease [6]. Maternally derived antibodies transferred with colostrum protect piglets from clinical disease shortly after weaning and may reduce virus transmission [6]. In Poland, two inactivated vaccines against influenza are licensed for pigs: Respiporc FLU3 (Ceva Santé Animale, Libourne, France) containing avH1N1 (1C.2), H3N2 and H1N2 (1B.1) virus antigens, for immunization of sows and gilts, and Respiporc FLUpan H1N1 (1A.3.3.2) (Ceva Santé Animale, Libourne, France) containing influenza A virus/Jena/VI5258/2009(H1N1)pdm09 for administration in pigs older than 8 weeks. The vaccine should be selected depending on the swIAV subtypes circulating in the herd. However, subtyping of swIAV is complex. There are three major IAV subtypes spread globally in pigs, H1N1, H3N2 and H1N2, but the origin and characteristics differ between geographic regions [3]. In Europe, H1N1, H3N2, H3N1 and H1N2 IAV subtypes circulate [7,8,9,10]. In 2009, the human pandemic IAV H1N1pdm09 was introduced, and this subtype became endemic in pigs in many countries.

The diagnosis of influenza involves laboratory detection of swIAV, as the clinical signs are not pathognomonic. Detection of the virus, its RNA or proteins, provides definitive evidence of the infection. The virus is most likely to be found in nasal secretions during the acute phase of infection [5]. Therefore, nasal swab (NS) samples have been used for swIAV PCR diagnosis and surveillance, but recent advances in the application of oral fluid (OF) for monitoring several swine diseases encouraged veterinarians to also exploit this material for the detection of swIAV. Several studies described swIAV detection in OF and showed advantages or disadvantages of this strategy [5,11,12,13]. Decorte et al. (2015) reported the detection of swIAV RNA at 21 days post-infection in 25% of OF samples, while nasal swabs reacted negatively by 7 days post-infection [5]. Our latest findings indicated that oral fluids contain sufficient amounts of intact swIAV RNA, enabling complete genome sequencing using the Oxford Nanopore Technologies platform [14].

As in most respiratory viral infections, seasonality has been demonstrated, and meteorological conditions are associated with swIAV survivability and transmissibility [15,16]. A cyclical pattern in IAV prevalence was observed by Pardo et al. (2017) [17]. swIAV levels can increase during fall, peak in both early winter (December) and late spring (May) and decrease in summer [17]. However, the mechanisms of correlation among swIAV prevalence, outdoor temperature and air humidity are not yet fully understood [17]. On the other hand, some studies documented a lack of seasonality of swIAV infections [8,18].

Continuously updated knowledge of swIAV strains circulating in pigs is of utmost importance for the diagnosis, prevention and control of influenza in pigs, but also to detect novel reassortant viruses that may pose a threat to human health. However, because of a mild course of endemic influenza, identification of infected animals for NS collection could be problematic in some cases. Therefore, PCR diagnosis and subtyping may be unsuccessful. OF sampling can provide a valid alternative with to the sampling of a larger proportion of the pigs [5]. The knowledge about the prevalence and the patterns of swIAV infections in Polish swine herds remains limited [7,8]. Thus, the aim of this study was to assess swIAV circulation patterns in Poland and improve protocols for efficient and cost-effective swIAV surveillance in pig farms.

## 2. Materials and Methods

### 2.1. Study Design and Sample Collection

The study was performed on samples collected from 2019 to 2021 in 35 Polish pig farms representing different sizes and types of production, where acute or mild influenza-like clinical signs were observed (Table S1). The samples from random pigs at the age of 5–20 weeks were collected by local veterinary practitioners. Ethical approval was waived for this study, as the analyzed materials originated from routine diagnostic investigations ordered by the farm owners. From each sampled age group, 4–5 NSs were obtained using the UTM^®^ system (COPAN Diagnostics Inc., Murrieta, CA, USA). One OF sample was obtained using cotton rope from the same pens as the NS. The samples were chilled and immediately transported to the laboratory to minimize the impact of the adverse conditions of sample handling and transport.

In nine of the above farms, influenza-like clinical signs were observed in cold months (October–April) and warm months (May–September), so the oral fluid samples were obtained twice during the study period. Additionally, due to the observation of respiratory signs, 13 farms were re-tested a few months later, following the primary sampling (Table S1). Porcine parainfluenza virus 1 (PPIV-1) and porcine reproductive and respiratory virus (PRRSV) statuses of tested farms are summarized in Table S1.

### 2.2. swIAV Detection and Molecular Subtyping

Before the nucleic acid extraction, NSs were pooled in groups of 4–5, and each pool corresponded to one pen of nursery pigs (5–12-week-old pigs) or fatteners (>12-week-old pigs). Next, the samples from swIAV-positive NS pools were tested individually. RNA was extracted using the IndiSPIN Pathogen Kit (Indical Bioscience GmbH, Leipzig, Germany) according to the manufacturer’s instructions. Extracted RNA was used for RT-PCR with *virotype* Influenza A RT-PCR Kit (Indical Bioscience GmbH, Leipzig, Germany) according to the manufacturer’s instructions. Subtyping of swIAV from individual NS samples was performed using multiplex real-time RT-PCR according to Henritzi et al. [19], where the following HA lineages were determined: H1pdm (H1A.3.3.2), H1av (1C.2) and H1hu (1B.1). The NA-only subtyping of N1 or N2 was performed with specific lineage determination, which was only possible for N1pdm (1A.3.3.2 origin) [19].

### 2.3. Statistical Analyses

Statistical analyses were performed using GraphPad Prism 8 for Windows (GraphPad Software, San Diego, CA, USA). The prevalence of swIAV in different diagnostic materials (NS and OF) and age groups (nursery pigs and fatteners) was compared using Fisher’s exact test. The comparison of swIAV Ct values and seasonality was made using the one-way ANOVA test. Comparison of Ct values in vaccinated and non-vaccinated pigs was performed using the Mann–Whitney test. A *p*-value < 0.05 was set as the statistically significant level.

## 3. Results

### 3.1. The Prevalence of swIAV in Herds with Influenza-like Clinical Signs

**Figure 1:**
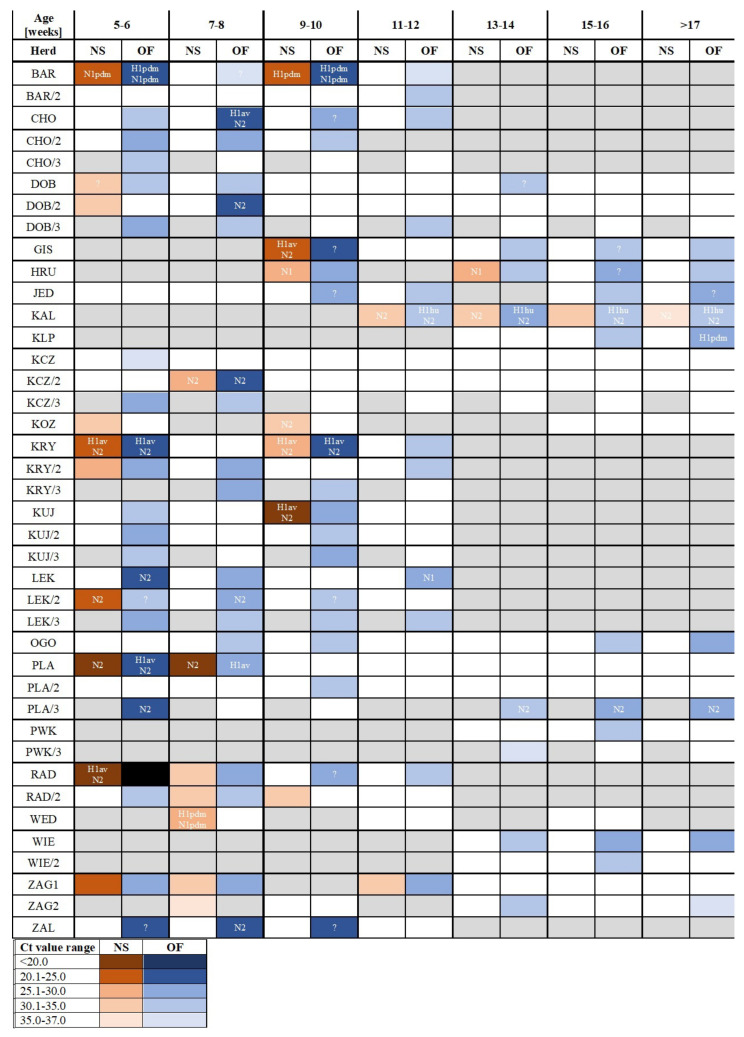
Detection of swine influenza A virus (swIAV) in different materials (nasal swab pool (NS) and oral fluid (OF)), age groups and samplings (-, 2, 3). Positive NS pools are marked with orange cells. Positive OFs are marked with blue cells. The negative pools are marked with white cells. The grey cells correspond to age groups that were not sampled. Positive samples were divided into five groups based on Ct values of the pool (<20.0; 20.0–25.0; 25.1–30.0; 30.1–35.0 and 35.0–37.0). Additionally, subtyping results are presented. A question mark in a cell indicates unsuccessful subtyping of a sample.

A farm was considered swIAV-positive if at least one sample of any kind, NS or OF, reacted positively in PCR for swIAV. SwIAV RNA was detected in 23 out of 35 (65.7%) tested farms: 10 independent ones and 13 that belonged to a large production system. In two farms (WED and KOZ), swIAV was detected only in NSs (Figure 1, Table S1), and in six farms, swIAV was detected only in OF samples (Figure 1, Table S1).

From swIAV-positive farms, 165 NS pools and 218 OFs were tested. Additionally, 35 (21.2%) NS pools and 106 (48.6%) OF samples were positive for swIAV. Ct values in NS pools (16.6–35.3; median = 29.4) and OFs (20.0–35.6; median = 28.4) were found to be similar (*p* > 0.05). The relative sensitivity of swIAV detection in a pen of pigs (in NS pools and OF) was calculated. Of the 34 pens where the virus was detected in NS pools, 88.2% of OFs (30) were positive. On the other hand, of the 84 pens where the virus was detected in OF, only 35.7% of NSs (30), pooled per pen, were positive.

In total, 40 and 19 samplings from 35 farms were performed in cold (October–April) and warm (May–SeptSeptember) months, respectively (Table S1). There was no significant difference (*p* > 0.05) in the swIAV prevalence on a herd level when comparing cold months (67.5%) to warm months (73.7%). The same was in the case for the pen-level prevalence in both NS and OF (NS: 17.3% vs. 11.1% and OF: 41.2% vs. 36.5%) in cold and warm months, respectively.

**Figure 2:**
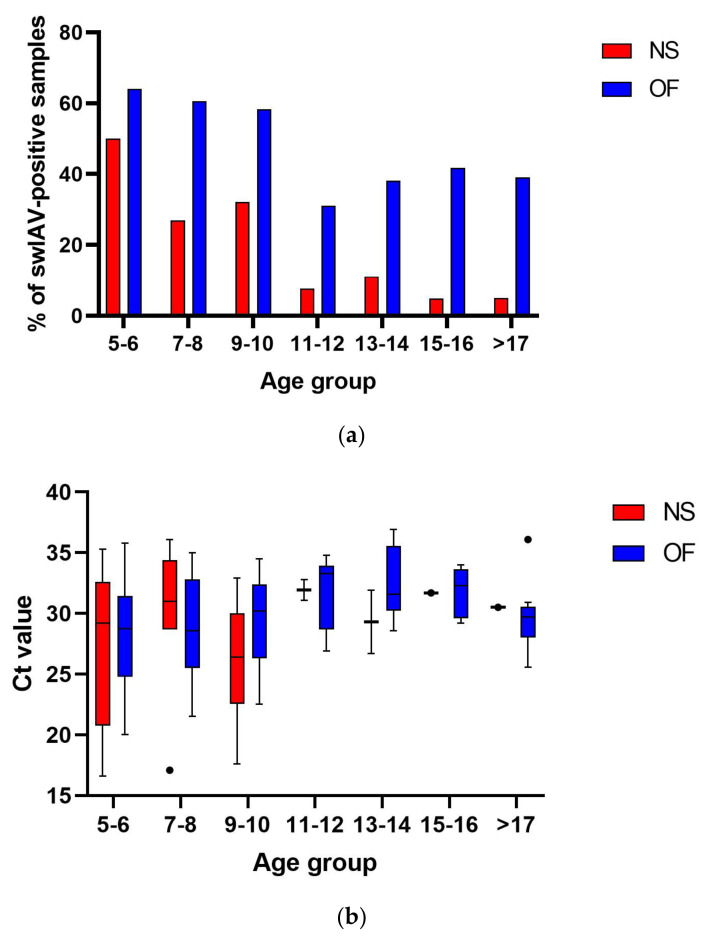
Percentages of swIAV-positive NS pools and OFs collected from different age groups of pigs (**a**). Comparison of swIAV Ct values in NS pools and OFs collected from different age groups of pigs (**b**). The whiskers plot uses a Tukey modification of whiskers. The comparison of swIAV Ct values was made using the one-way ANOVA test. No statistically significant differences were observed in Ct values between age groups.

The prevalence of swIAV in NS pools was significantly (*p* < 0.05) higher in nursery pigs (31 out of 106, 29.2%) than in fatteners (4 out of 59, 6.8%). No differences were found in Ct values between these groups (16.6–36.1; median = 29.5 and 26.7–31.9; median = 31.1 for nursery pigs and fatteners, respectively). The proportion of swIAV-positive age groups differed between the farms. Overall, 5-week-old piglets were the group with the highest swIAV prevalence (13 out of 26 NS pools tested, 50.0%) (Figure 2), while fatteners from only two farms, KAL and HRU, were swIAV-positive (Figure 1).

Testing individual NS samples from qPCR-positive pools showed different proportions of swIAV-positive pigs in one pen, and it ranged from 100.0% (e.g., farm KAL, KRY, PLA, KOZ) to only one swIAV-positive NS (e.g., farm HRU or BAR) (Table S2). The Ct values of individual NSs differed between pigs from the same pen, e.g., in farm PLA, all 5-week-old piglets were swIAV-positive, and the Ct values ranged from 15.9 to 33.8 (Table S2).

No statistically significant differences (*p* > 0.05) between the swIAV prevalence in OF collected from nursery pigs (79 out of 150, 52.7%) and fatteners (27 out of 68, 39,7%) were found. However, Ct values were significantly lower (*p* < 0.05) in nursery pigs (20.0–35.8; median = 29.6) than in fatteners (25.6–36.9; median = 33.6). Similarly to the detection in NSs, the highest swIAV prevalence (64.1%) with the lowest Ct values (20.0–35.8; median = 28.8) was found in 5–6-week-old piglets (Figure 2).

**Figure 3:**
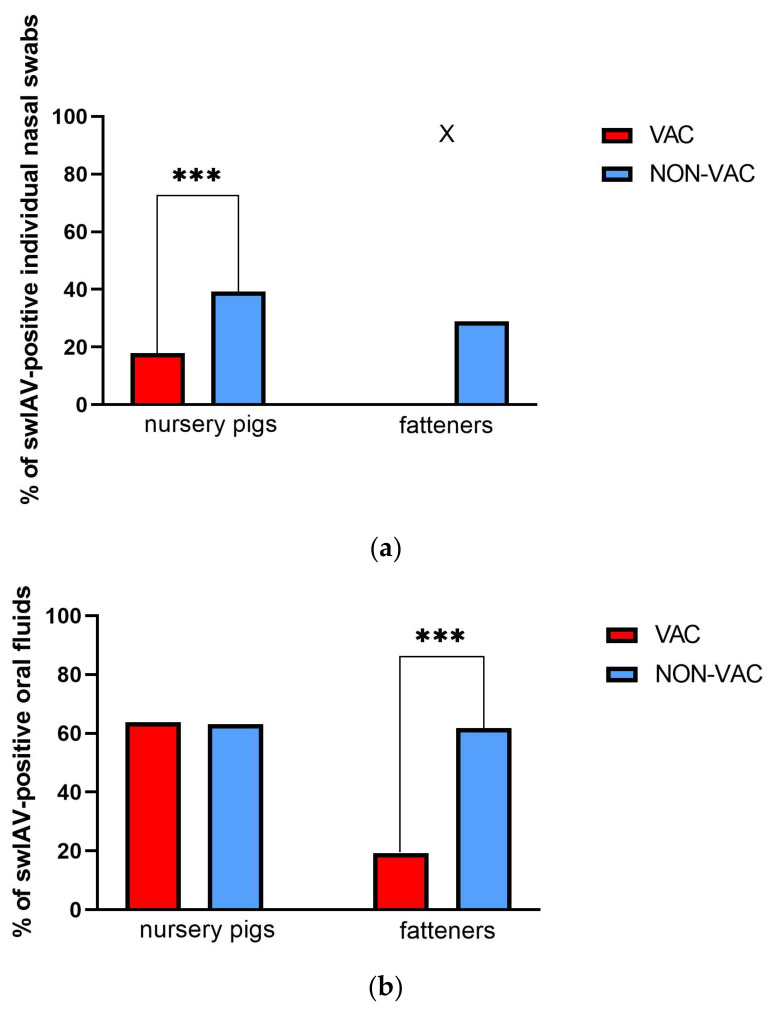
Comparison of swIAV detection in samples collected from nursery pigs of sows vaccinated with Respiporc FLU3 (VAC) and non-vaccinated sows (NON-VAC). Percentages of swIAV-positive individual nasal swabs in nursery pigs (5–12 weeks old) and fatteners (>12 weeks old) in individual nasal swabs (**a**) and oral fluids (**b**). SwIAV prevalence was compared using the Fisher test. There were no significant differences (*p* > 0.05 in Mann–Whitney test) in Ct values between tested groups. “X” means that statistical comparison was not performed. *p* < 0.001 was marked with ***.

In 14 of 23 swIAV-positive farms, sows were vaccinated two weeks before with the Respiporc FLU3 (CEVA) vaccine (VAC) (Table S1). Pigs from nine swIAV-positive farms were from sows not vaccinated against influenza (NON-VAC). Comparison of virus circulation indicated that individual NSs of nursery pigs born to influenza vaccinated and unvaccinated sows showed 17.9% (43 out of 240) prevalence in the former group and 39.3% (35 out of 89) in the latter group (Figure 3). This difference was statistically significant (*p* < 0.05) (Figure 3). Ct values in nursery pigs were 15.9–36.1; median = 30.1 for vaccinated and 18–36.1; median = 31.8 for non-vaccinated ones. Interestingly, swIAV prevalence in OF was almost the same in vaccinated and non-vaccinated nursery pigs (63.8% and 63.2%, respectively) with Ct values 20–35.8; median = 29.5 and 22.5–34.5; median = 28.9, respectively.

Comparison in fatteners showed that in both materials, virus prevalence was higher in the NON-VAC group, where swIAV was found in 28.9% (26 out of 90) of individual nasal swabs (Ct = 27.7–36.6; median = 31.2) and 61.9% (13 out of 21) of OF (Ct = 26.9–36.2; median = 31.8) (Figure 3).

Ct values in individual nasal swabs and oral fluids were similar (*p* > 0.05) in both nursery pigs and fatteners.

### 3.2. Subtyping of swIAV from Polish Pig Herds

SwIAV from 51 NS and 38 OF samples from 16 herds were typed using multiplex RT-qPCR according to Henritzi et al. [19]. swIAV NA determination using the above method was successful for 45.1% of NS and 60.5% of OF. HA determination was less successful, and subtype was determined only in 21.6% of NSs and 31.6% of OFs. Determination of both NA and HA was possible only in six NS (11.8%) and 10 OF (26.3%) samples.

Results of subtyping showed that N2 was the most prevalent and was found in 11 herds. In farm LEK, N2 and N1 were identified in OFs collected from 5- and 11-week-old pigs, respectively. H1av was found in six herds (Figure 1). Subtyping showed that H1pdm09N1pdm09 was detected in two herds (BAR and WED) (Figure 1). Interestingly, H1huN2 was found in all tested OFs from herd KAL.

## 4. Discussion

Infectious disease control requires fine-tuning of diagnostic and monitoring protocols. This is particularly important for endemic diseases whose clinical signs are not pathognomonic, such as influenza in swine. The knowledge of pathogen circulation and prevalence in the population and the changes when introducing a control program may aid in determining the effect of applied measures.

Real-time RT-PCR testing of NSs is most commonly applied for swIAV diagnosis. SwIAV prevalence on a herd level assessed by NS testing was found to vary between European countries. Simon et al. (2014) reported that 2759 out of 9025 (31.0%) tested herds from 14 European countries were considered swIAV-positive based on RT-PCR results, and the prevalence ranged from 0% in Lithuania to 67.0% in Russia [7]. High swIAV prevalence was also detected in France (433 out of 818, 53%) and the Netherlands (42 out of 87, 48.0%) [7]. Higher swIAV detection rates at the country level were described by Henritzi et al. (2020), where 56.6% of 2457 tested herds were swIAV-positive and the prevalence ranged from 0% in Austria and Sweden to >65% in the Netherlands, Denmark, Spain, the Republic of Ireland, Slovakia and Switzerland [8]. The above-mentioned studies also described swIAV detection in Poland. In one, 56 out of 185 tested herds (30%) were swIAV-positive by PCR, but only herd-level swIAV prevalence was assessed [7]. In another study, swIAV prevalence in Polish herds was found to be 46.2%, but samples (mainly NS) from only 13 pig farms were tested [8]. In the present study, swIAV was detected in 63.9% of farms, which was a much higher proportion than previously reported. However, unlike in the previous reports, which dealt with clinical samples from affected pigs, in the current study, multiple age groups from each herd were tested to better understand swIAV circulation in large pig populations. Variations between the prevalence detected in different countries and studies may reflect the differences in the virus circulation between them. Still, they can also be the result of different NS sampling techniques. Personal and veterinary practitioners’ observations showed that NS sampling quality has a significant influence on the success of swIAV detection as well as subtyping.

Control of swIAV in farrow-to-wean farms is a crucial factor in minimizing the virus’s spread across geographical regions, since infected piglets could transmit swIAV to other farms [6]. Several studies described patterns of swIAV circulation in farrow-to-finish or farrow-to-wean farms [2,20,21]. However, these studies were conducted in single or in few herds [12,21]. The results of the present study on post-weaning pigs largely confirm earlier observations that swIAV infections are more frequent in young piglets than in older groups [6,22], and the virus was most prevalent in 5-week-old piglets. Interestingly, testing individual NSs showed different proportions and a varying range of Ct values in the diagnosed age groups, regardless of herd vaccination status (Table S2).

In the present study, pairs of NS pools and OF representing the same pens of pigs were examined. In the vast majority of cases of testing pigs from the same pen, swIAV detection in NS pools was confirmed using OF, and Ct values were similar in both materials. A similar observation was made by Decorte et al. (2015) in experimentally infected pigs [5]. However, in four pens from three farms, where swIAV nasal shedding (Ct > 30.5) was identified, the virus was not detected in OF (Figure 1). This discrepancy might be explained by a very early stage of swIAV infection.

Any virus detection in OF samples should be analyzed with caution. SwIAV detection in NSs can be interpreted as an ongoing infection in at least the upper respiratory tract and may be related to some clinical manifestation in the sampled population [4,5]. On the other hand, swIAV can be present in OF of acutely infected, diseased pigs and asymptomatic convalescent pigs [5]. Environmental contamination could also lead to such findings. It was shown that swIAV RNA could be detected in oral fluids at the same time as in NSs and a few weeks after nasal shedding ceased [5]. It was indicated that the probability of detecting swIAV in OF by RT qPCR was equal to NSs until 6 days post-infection and higher in the following days [5]. In this study, only 35.7% of cases of swIAV detection in OF were confirmed in NS pools. On the other hand, the sensitivity of OF was much higher and amounted to 88.2%. The finding of swIAV only in OF samples, in the absence of the virus detection in NS from tested farms of this, may also indicate a low prevalence of ongoing infection, which the employed protocol of NS collection and testing was not able to detect. In addition, as more pigs contribute to an OF sample compared to the NS pool (only representing 4–5 pigs), the likelihood of finding swIAV is higher in OF. Moreover, a similar prevalence of swIAV in OF collected from nursery pigs and fatteners may suggest that this material is optimal for fatteners, and testing only NS pools may lead to underestimating the role of swIAV in this age group of pigs. In summary, OF could be a sample material used for cost-effective monitoring of swIAV presence in a farm. An examination of OF could be recommended for the confirmation of swIAV, even when flu-like influenza signs have vanished.

As mentioned previously, swIAV subtyping is complex. This study showed that swIAV subtyping is more effective in OF. However, the success rate of IAV subtyping did not depend on the Ct value of NS or OF samples. Despite many swIAV-positive samples, in only six NS (11.8%) and 10 OF (26.3%) samples, both NA and HA were subtyped. The highest H1av lineage prevalence is in accordance with previous European studies carried out in 2015–2018, where it was detected in 54.2% of farms [8]. Interestingly, in the present study, the reassortant H1avN2 was found to be most prevalent, while previous studies described its sporadic cases in Poland [8]. Detection of N2 and N1 in OFs collected from LEK indicated that two different swIAV strains circulated in the farm. Preliminary subtyping performed in 2010–2013 showed that in most European countries H1N1pdm09 was one of the dominating swIAV subtypes [7]. Interestingly, in our study, H1pdmN1pdm was found in two independent herds (BAR and WED) located in different districts of Poland. H1pdm was detected in farm KLP, which belongs to the same company as farm BAR. The occurrence of various swIAV genotypes indicates the need to update knowledge about their circulation in the Polish pig population. However, it seems that the subtyping protocol proposed in this study was not efficient, and because of the high genetic diversity of the strains, there is still a need to update the sequences of primers.

It was shown that sow vaccinations or infections leading to high swIAV-specific MDA may reduce swIAV circulation in suckling piglets and nursery pigs [6]. However, assessing the effectiveness of vaccination in a herd seems problematic. Our findings showed that the testing of swIAV prevalence in NSs of nursery pigs could be useful to evaluate vaccination efficacy since in Polish herds, where vaccination against swIAV was performed in sows, a statistically significant (*p* < 0.05) reduction of swIAV prevalence in individual NSs was found (Figure 3). However, the detection of swIAV in OF collected from nursery pigs was similar in vaccinated and non-vaccinated herds, underlining that swIAV vaccinations using the currently available vaccines cannot eliminate swIAV from the herds. The effect of sow vaccination is mainly expected in piglets. However, in this study, a significant reduction of swIAV detection was found in OF collected from fatteners originating from vaccinated sows. Statistical analyses were waived in individual NSs collected from fatteners since in the vaccinated group all of these samples were negative; however, the difference in prevalence is visible (0% vs. 28.9% in vaccinated and non-vaccinated farms, respectively). It should be noted that pigs reared from vaccinated sows belonged to a large multisite production system. This, together with a higher level of biosecurity and overall hygiene, could also impact virus prevalence. Therefore, the true role of vaccination in limiting swIAV prevalence in nursery and growing pigs cannot be unequivocally defined in the current study.

Improving swIAV diagnostics is still a challenge. The experience we propose showed that OF may be useful for monitoring swIAV infections and subtyping. However, this specimen type cannot replace testing NSs, which are useful in the assessment of swIAV vaccination effects in nursery pigs. swIAV subtyping protocols need improvement. There is still a gap in knowledge about the genetic variability of swIAV, especially in Eastern Europe. Thus, further studies are needed to evaluate swIAV genetic diversity in Poland.

## Data Availability

The data presented in this study are available on request.

## Supplementary Materials

The following supporting information can be downloaded at https://www.mdpi.com/article/10.3390/pathogens14080808/s1, Table S1: Farm overview; Table S2: IAV Ct values of individual nasal swabs in different age groups.

## Abbreviations

The following abbreviations are used in this manuscript:

IAV	influenza A virus
swIAV	swine influenza A virus
NS	nasal swab
OF	oral fluid
HA	hemagglutinin
NA	neuraminidase
PRDC	porcine respiratory disease complex
MDA	maternally derived antibody
PRRSV	porcine reproductive and respiratory syndrome
PPIV-1	porcine parainfluenza virus 1
