# Supplementary material for: Can Oral Fluids Replace Nasal Swabs in Swine Influenza A Virus (swIAV) PCR Diagnostics?
Source: Pathogens. 2025 Aug 14;14(8):808. doi: 10.3390/pathogens14080808 (PMC12389440; doi:10.3390/pathogens14080808)
Supplement: Supplementary file 1 [file pathogens-14-00808-s001.zip › Table S1.pdf]

Farm ID	Ownership	Type of production	Sows vaccination against IAV	Localization		% of IAV-positive samples (number of positives/number of tested)								Herd status	
				Province	District	Sampled in	NS	OF	Sampled in	OF	Sampled in	NS	OF	PPIV-1	PRRSV
NW	independent	Farrow-to-finish	No	Lubusz	wschowski	**Oct 2020**	0.0 (0/35)	0.0 (0/7)						Neg	?
BAR	B	Nursery	Yes	West Pomeranian	gryfiński	**Feb 2019**	15.0 (3/20)	100.0 (4/4)			**Jul 2021**	0.0 (0/35)	14.3 (1/7)	Pos	Neg
KLP	B	Fattening	Yes	Greater Poland	złotowski	**Apr 2019**	0.0 (0/15)	66.6 (2/3)	**Jul 2019**	0.0 (0/3)	**Jul 2020**	0.0 (0/15)	0.0 (0/3)	Pos	Neg
KOCZ	B	Multiplicating farm	Yes	Pomeranian	człuchowski	**Feb 2019**	0 (0/35)	14.3 (1/7)	**Jul 2019**	28.6 (2/7)	**Jun 2020**	5.7 (2/35)	14.3 (1/7)	Pos	Neg
KRY	B	Nursery	Yes	Greater Poland	nowotomyski	**Feb 2019**	15.0 (3/20)	75.0 (3/4)	**Jul 2019**	50.0 (2/4)	**Aug 2020**	5.0 (1/20)	75.0 (3/4)	Pos	Neg
PLA	B	Multiplicating farm	Yes	Pomeranian	człuchowski	**Jan 2019**	20.0 (7/35)	28.6 (2/7)	**Sep 2019**	66.6 (4/6)	**Jul 2021**	0.0 (0/35)	14.3 (1/7)	Pos	Neg
PWK	B	Fattening	Yes	Pomeranian	człuchowski	**Feb 2019**	0 (0/15)	33.3 (1/3)	**Jul 2019**	33.3 (1/3)	**May 2020**	0.0 (0/15)	0.0 (0/3)	Pos	Neg
ZAL	B	Nursery	Yes	Pomeranian	człuchowski	**Feb 2019**	0.0 (0/20)	75.0 (3/4)			**Mar 2020**	5.0 (1/20)	100.0 (4/4)	Pos	Neg
DOB	B	Multiplicating farm	Yes	Pomeranian	człuchowski	**Feb 2019**	8.6 (3/35)	42.9 (3/7)	**Jul 2019**	42.9 (3/7)	**Jun 2020**	2.9 (1/35)	14.3 (1/7)	Pos	Neg
KUJ	B	Nursery	Yes	Pomeranian	człuchowski	**Jan 2019**	10.0 (2/20)	50.0 (2/4)	**Jul 2019**	50.0 (2/4)	**Oct 2021**	0.0 (0/20)	75.0 (3/4)	Pos	Neg
LEK	B	Nursery	Yes	Pomeranian	człuchowski	**Jan 2019**	0.0 (0/20)	75.0 (3/4)	**Jul 2019**	100.0 (4/4)	**Mar 2020**	11.4 (4/35)	75.0 (3/4)	Pos	Neg
RAD	B	Nursery	Yes	West Pomeranian	łobeski	**Apr 2019**	30.0 (6/20)	100.0 (4/4)			**Jul 2020**	0.0 (0/20)	100.0 (3/3)	Pos	Neg
CHO	B	Nursery	Yes	West Pomeranian	choszczeński	**Apr 2019**	0.0 (0/20)	100.0 (4/4)	**Oct 2019**	25.0 (1/4)	**Aug 2020**	0.0 (0/15)	100.0 (3/3)	Pos	Neg
CZA	B	Fattening	Yes	West Pomeranian	białogardzki	**Apr 2019**	0.0 (0/15)	0.0 (0/3)			**Aug 2020**	0.0 (0/15)	0.0 (0/3)	Pos	Neg
NAC	B	Fattening	Yes	West Pomeranian	koszaliński	**Mar 2019**	0.0 (0/15)	0.0 (0/3)						Pos	Neg
UNI	B	Fattening	Yes	Pomeranian	człuchowski	**Feb 2019**	0.0 (0/15)	0.0 (0/3)						Pos	Neg
SYP	B	Multiplicating farm	Yes	Kuyavia-Pomeranian	sępoleński	**Feb 2019**	0.0 (0/35)	0.0 (0/7)						?	Neg

SWI	B	Fattening	Yes	West Pomeranian	koszaliński	**Mar 2019**	0.0 (0/15)	0.0 (0/3)						Neg	Neg
WIE	B	Fattening	Yes	West Pomeranian	walecki	**Mar 2019**	0.0 (0/15)	100.0 (3/3)						?	Neg
STA	independent	Farrow-to-finish	No	Kuyavia-Pomeranian	lipnowski	**Feb 2020**	0.0 (0/25)	0.0 (0/4)						Pos	?
WED	independent	Farrow-to-finish	No	Silesian	lubliniecki	**Mar 2019**	17.5 (7/40)	0.0 (0/6)						Pos	?
ZAG1	independent	Farrow-to-finish	No	Lodz	tomaszowski	**Nov 2019**	31.4 (11/35)	42.9 (3/7)						Pos	?
ZAG2	independent	Farrow-to-finish	No	Lodz	tomaszowski	**Nov 2019**	5.7 (2/35)	50.0 (2/4)						Pos	?
TUB	independent	Farrow-to-finish	No	Lodz	tomaszowski	**Nov 2019**	0.0 (0/30)	0.0 (0/6)						Pos	?
JAN	independent	Farrow-to-finish	No	Mazovian	miński	**Feb 2020**	0.0 (0/20)	0.0 (0/3)						Pos	?
SRC	independent	Farrow-to-finish	No	Opole	oleski	**Mar 2019**	0.0 (0/25)	0.0 (0/7)						Pos	Pos
KAL	independent	Farrow-to-finish	No	Lodz	zgierski	**Dec 2018**	95.0 (19/20)	100.0 (6/6)						neg	?
KOZ	independent	Farrow-to-finish	No	Mazovian	sokołowski	**Feb 2019**	20.0 (10/50)	0.0 (0/5)						Neg	Pos
KN1	independent	Farrow-to-finish	No	Opole	kluczborski	**Mar 2019**	0.0 (0/25)	0.0 (0/5)						Neg	Pos
KN2	independent	Farrow-to-finish	No	Opole	kluczborski	**Mar 2019**	0.0 (0/25)	0.0 (0/5)						Neg	Pos
ZYW	independent	Nursery	No	Lodz	piotrkowski	**Nov 2019**	70.0 (17/20)	100.0 (4/4)						neg	Pos
OGO	independent	Farrow-to-finish	No	Mazovian	plocki	**Apr 2019**	0.0 (0/35)	57.1 (4/7)						?	?
HRU	independent	Fattening	No	Podlaskie	losicki	**Apr 2019**	10.0 (2/20)	50.0 (2/4)						?	?
GIS	independent	Fattening	No	Silesian	gliwicki	**Apr 2019**	10.0 (2/20)	80.0 (4/5)						?	?
JED	independent	Farrow-to-finish	Yes	?	?	**Mar 2019**	0.0 (0/30)	66.6 (4/6)	**Jul 2019**	0.0 (0/6)				?	?

Supplementary table 1: Overview on tested herds. A question mark indicates data not availiable.
