# Supplementary material for: Can Oral Fluids Replace Nasal Swabs in Swine Influenza A Virus (swIAV) PCR Diagnostics?
Source: Pathogens. 2025 Aug 14;14(8):808. doi: 10.3390/pathogens14080808 (PMC12389440; doi:10.3390/pathogens14080808)
Supplement: Supplementary file 1 [file pathogens-14-00808-s001.zip › Table S2.pdf]

Farm ID	Age [weeks]						
	5-6	7-8	9-10	11-12	13-14	15-16	17-18
DOB							
	31.6						
	28.6						
	33.7						
PLA	15.9	17.9					
	33.8						
	32.3						
	31.2	20.4					
	21.7						
ZAG1	18.2			33.4			
				32.5			
	34.7	34.1		35.1			
	34.3			31.8			
	28.2	31.9		32.3			
ZAG2		29.4					
		28.6					
WED		33.8					
		29.4					
		33.2					
KAL				30.9	31.3	31.0	33.9
				31.6	30.5	31.0	28.6
				32.1	31.6	33.4	31.5
				29.1	29.6	30.8	32.9
				30.6	31.6	28.9	36.6
GIS							
			32.9				
			23.5				
HRU			27.4				
					25.7		
KOZ	29.4		31.4				
			28.2				
	32.4		32.6				
			32.5				
	28.8		28.6				
BAR							
	29.2						

	33.5		24.3				
			18.6				
KUJ			17.7				
	28.8						
	30.8	32.6					
RAD	19.3						
	31.7						
	26.3						
			36.1				
	25.8		35.6				
KRY	25.2		28.9				
			22.5				
			31.5				
	31.9						
	22.1		30.1				
	31.9						
	31.2						
ZYW	31.5						
			32				
			29.6				
	34.9		34				
			36.1				
	34.7		30.5				
		25.6					
KOCZ/2							
		31.1					
	27.1						
KRY/2	34.21						
		35.8					
RAD/2		30.1					
			31.3				
			34.9				
	35.9						
LEK/2	35.6						
	20.3						
	34.3						
ZAL/2	35.7						

DOB/2	29.1						

Table S2: swIAV Ct values of individual nasal swabs in different age groups. The grey cells correspond to age groups that were not sampled.
